# The influence of stabilizers on the production of gold nanoparticles by direct current atmospheric pressure glow microdischarge generated in contact with liquid flowing cathode

**DOI:** 10.1007/s11051-015-2992-7

**Published:** 2015-04-17

**Authors:** Anna Dzimitrowicz, Piotr Jamroz, Krzysztof Greda, Piotr Nowak, Marcin Nyk, Pawel Pohl

**Affiliations:** Faculty of Chemistry, Wroclaw University of Technology, Wybrzeze Stanislawa Wyspianskiego 27, 50-370 Wroclaw, Poland

**Keywords:** Gold nanoparticles, Atmospheric pressure glow discharge, UV/Vis absorption spectroscopy, Localized surface plasmon resonance

## Abstract

Gold nanoparticles (Au NPs) were prepared by direct current atmospheric pressure glow microdischarge (dc-μAPGD) generated between a miniature argon flow microjet and a flowing liquid cathode. The applied discharge system was operated in a continuous flow liquid mode. The influence of various stabilizers added to the solution of the liquid cathode, i.e., gelatin (GEL), polyvinylpyrrolidone (PVP), or polyvinyl alcohol (PVA), as well as the concentration of the Au precursor (chloroauric acid, HAuCl_4_) in the solution on the production growth of Au NPs was investigated. Changes in the intensity of the localized surface plasmon resonance (LSPR) band in UV/Vis absorption spectra of solutions treated by dc-μAPGD and their color were observed. The position and the intensity of the LSPR band indicated that relatively small nanoparticles were formed in solutions containing GEL as a capping agent. In these conditions, the maximum of the absorption LSPR band was at 531, 534, and 535 nm, respectively, for 50, 100, and 200 mg L^−1^ of Au. Additionally, scanning electron microscopy (SEM) and dynamic light scattering (DLS) were used to analyze the structure and the morphology of obtained Au NPs. The shape of Au NPs was spherical and uniform. Their mean size was ca. 27, 73, and 92 nm, while the polydispersity index was 0.296, 0.348, and 0.456 for Au present in the solution of the flowing liquid cathode at a concentration of 50, 100, and 200 mg L^−1^, respectively. The production rate of synthesized Au NPs depended on the precursor concentration with mean values of 2.9, 3.5, and 5.7 mg h^−1^, respectively.

## Introduction

Recently, a special attention has been paid to the synthesis of gold nanoparticles (Au NPs) that may be used in medicine (Butle and Baheti 2011), genomics (Larguinho and Baptista 2012), biology (Heddle 2013), cosmetology (Saha et al. 2011), and optics (Fischer et al. 2012) due to their respective chemical (Giljohann et al. 2010), physical (Xia et al. 2009), therapeutic (Lan et al. 2013), and electronic (Bastús et al. 2011) properties.

Usually, for the production of Au nanostructures, the reverse micelles technique (Eastoe et al. 2006), sonochemical (Okitsu et al. 2005), chemical (Jana et al. 2001; Sivaraman et al. 2010), and photochemical (Lafon et al. 2012) reduction methods were applied. In contrast to those conventional methods, plasma-based methods were less explored and used for the synthesis of Au NPs (Chen et al. 2012; Chiang et al. 2010; Huang et al. 2014; Mariotti et al. 2012; Richmonds and Sankaran 2008; Patel et al. 2013; Shirai et al. 2014; Vlad et al. 2014). Among different plasma systems, atmospheric pressure microdischarge generated between gaseous microjets and different liquid cathodes is established as a very promising method of the synthesis of NPs due to its one-stage character and simplicity, low operational costs, and the lack of toxic by-products (Chiang et al. 2010; Mariotti et al. 2012). Unfortunately, all microdischarge systems cited above were working in a non-flowing, stationary mode.

Recently, Shirai et al. (2014) have investigated the synthesis of Au NPs using a dual atmospheric pressure glow microdischarge system, in which two He microjets were in contact with liquids. They found that Au NPs were generated in both discharge zones, i.e., in cathodic and anodic compartments. In another work, Patel et al. (2013) produced Au NPs in a system, in which APGD was generated between a He microjet and a bulky liquid cathode. Gold nanostructures of different morphologies were synthesized in aqueous solutions. The size of the obtained NPs occurred to be in the range from five to several hundreds nm. The effect of selected experimental conditions, including the discharge current, the solution temperature as well as the stirring of the solution, was studied by Huang et al. (2014) for microdischarge generated between He microjet and a bulky liquid cathode. These researchers found that investigated parameters affected the size distribution of Au NPs.

Unfortunately, just a little attention has been paid to the use of stabilizers in the synthesis of NPs using microdischarges generated in contact with liquid. Only a few relevant works reported results about the effect of the addition of stabilizers to solutions of liquid cathodes in order to produce small-sized Au NPs (Richmonds and Sankaran 2008; Chen et al. 2012). Accordingly, Richmonds and Sankaran (2008) established that the kinetic growth of Au NPs was related to the concentration of fructose, added to stabilize nanostructures formed. This capping agent prevented uncontrolled particle growth and agglomeration; hence, Au NPs with the mean size equal to 10 nm were obtained. The effect of DNA as a stabilizer on the production of Au NPs was also studied by Chen et al. (2012) in a microplasma system generated in contact with a bulky solution containing HAuCl_4_ as an Au NPs precursor. Those authors found that DNA prevented the aggregation and the sedimentation of Au NPs and their uncontrolled growth. The size distribution of Au NPs as well as their morphology was found to be related to concentrations of HAuCl_4_ and DNA in the solution. The mean size of Au NPs was changed from 6 to 24 nm.

The main objective of the present work was to evaluate the suitability of a new direct current atmospheric pressure glow microdischarge (dc-μAPGD) system with a continuous flow of solutions, acting as the liquid cathode of the discharge system, to synthesize Au NPs. In addition, the effect of different stabilizers added to solutions on granulometric properties of resultant Au NPs was compared. HAuCl_4_ was used as the Au NPs precursor, while gelatin, polyvinylpyrrolidone, and polyvinyl alcohol were applied as capping agents. Optical properties of solutions treated by dc-μAPGD and containing Au NPs were measured using ultraviolet–visible (UV/Vis) absorbance spectroscopy. The morphology and the size of Au NPs were characterized by scanning electron microscopy and dynamic light scattering. Possible reactions taking place in interfacial and liquid zones of the discharge were briefly discussed.

## Experimental section

### Synthesis of Au NPs

**Fig. 1:**
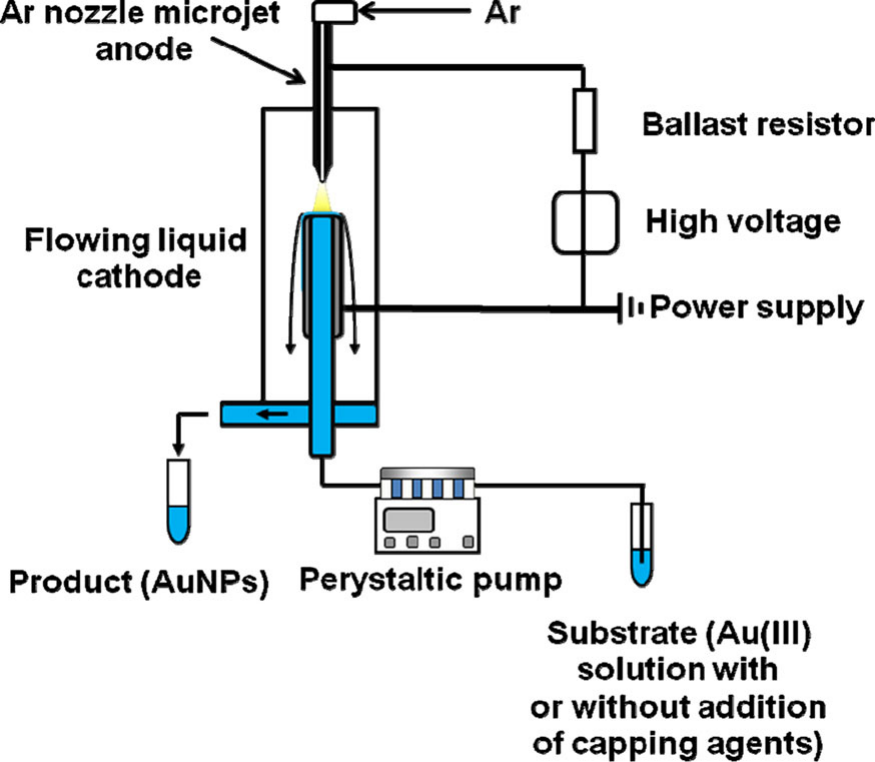
The experimental setup (not to scale)

Au NPs were synthesized in a miniaturized reactor based on dc-μAPGD sustained in a discharge system given in Fig. 1. The reactor was working in a liquid flowing mode without the re-circulation and stirring of solutions. dc-μAPGD was ignited between an Ar nozzle microjet working as an anode and a liquid flowing cathode being a HAuCl_4_ solution acidified with HCl to a final concentration of 0.1 mol L^−1^. This concentration of HCl was optimal and kept constant to provide repeatable discharge conditions. Lower than 0.1 mol L^−1^ or higher than 1.0 mol L^−1^ concentrations of HCl were also tested but established insufficient for the stability of the discharge in reference to the cathode fall and the current of the discharge. In addition, higher concentrations of HCl could have a negative leaching effect on Au NPs formed. The flow rate of solutions of the liquid cathode introduced to the system was 1.5 mL min^−1^. High-purity Ar (99.996 %, Messer, Poland) was introduced through a stainless steel nozzle (ID 500 μm) at a miniature flow rate, controlled by a Tylan General flow controller (FC-2900), and a digital meter (RO-28). The steel nozzle enabled to form the gaseous microjet. A dc-HV generator (Dora, Poland) was applied for the ignition of the discharge and its operation. A two-channel peristaltic pump (LabCraft, France) was used to deliver solutions to the microdischarge system through a quartz tube (ID 2.0 mm) with an imposed graphite tube (ID 4.0 mm) at the end. A Pt wire attached to the graphite tube provided the electric contact to solutions of the liquid flowing cathode. Overflowing the graphite–quartz tube, these solutions were collected in a small reservoir and continuously drained by the peristaltic pump to plastic tubes (collectors). A ballast resistor (10 kΩ, Tyco Electronics, USA) was applied in the anode circuit to stabilize the discharge current. The nozzle and the graphite–quartz tube were vertically arranged and the distance between them was 5.0 mm. dc-μAPGD was sustained and stably operated after passing through the nozzle—a miniature flow of Ar (150 sccm at STP)—and applying a voltage of 1100–1300 V. This resulted in a flow of the discharge current of 40 mA.

### Reagents and solutions

All reagents used for synthesis were of analytical grade. Re-distilled water was used throughout. A stock solution of Au (1000 mg L^−1^) was prepared by dissolving HAuCl_4_ × 3H_2_O (Merck, Germany) in re-distilled water. Working solutions of Au at concentrations of 50, 100, and 200 mg L^−1^ were prepared from the stock solution by appropriate dilutions with water. The conductivity of all solutions of Au was adjusted with a concentrated 37 % (m/m) HCl solution (Avantor Performance Materials, Poland). The final concentration of HCl in these solutions was 0.10 mol L^−1^. To prevent the agglomeration and the sedimentation of Au NPs, GEL (average molecular weight 80,000 g mol^−1^, 99.9 % purity, Rousselot, USA), PVP (average molecular weight 40,000 g mol^−1^, 99.9 % purity, Sigma-Aldrich, Poland), and PVA (average molecular weight 89,000–98,000 g mol^−1^, 99.9 % purity, Sigma-Aldrich, Poland) were added to solutions. Final concentrations of mentioned stabilizers in these solutions were 0.5 % (m/v).

### Characterization and purification of Au NPs

All solutions treated by the microdischarge in different experimental conditions were analyzed to confirm the formation of Au NPs, estimate their optical properties as well as the morphology and the size of nanostructures.

The kinetics growth of Au NPs was monitored by UV/Vis absorbance spectroscopy (Huang et al. 2014; Richmonds and Sankaran 2008) using an Ocean Optics USB-4000-UV/Vis spectrometer with optical fibers (SMA 905 numerical aperture single-strand fibers), a deuterium tungsten halogen lamp, and a 4-way cuvette holder (Ocean Optics, USA) and a standard 1-cm quartz cuvette. The integration time used for UV/Vis measurements was 10 s. Absorption spectra were carried out in the range of 200–800 nm with a step of 1 nm. The absorption spectrum of de-ionized water acidified with HCl (0.1 mol L^−1^) was used as a background reference. Additionally, the absorption spectra of initial solutions, loaded to the dc-μAPGD system, were recorded. Optical properties of solutions treated by dc-μAPGD were directly measured in a function of a post-microdischarge treatment time up to 1440 min.

For the appointment of the morphology of nanostructures obtained, solutions were purified to remove non-Au NPs components and collected by centrifugation at 12,000 rpm, for 10 min in a MPW-350 centrifuge (MPW Medical Instruments, Poland). There were three rounds of Au NPs collection and purification. Au NPs were purified at first using centrifugation and decantation of 5 mL of a freshly synthesized Au NPs solution. The supernatant resulting from the initial centrifugation was transferred to a centrifuge tube. The supernatant was subjected to a second and a third round of centrifugation by following the same operating procedure as followed in the first round to minimize the influence of the stabilizer and obtain a purified Au NPs solution. The purified Au NPs obtained in three steps were immediately dispersed in de-ionized water of volume 5 mL and then separated by centrifugation.

The production rate of Au NPs (in mg h^−1^) was calculated on the basis of concentrations of Au NPs in a solution and in relation to the flow rate of the liquid cathode (1.5 mL min^−1^). For this purpose, the portion of a solution treated by dc-μAPGD was transferred to a centrifuge tube. Au NPs were centrifuged (10 min at 12,000 rpm), and the obtained Au NPs were washed using de-ionized water and then separated by centrifugation. Purified Au NPs were immediately digested in 2.0 mL of a concentrated *aqua regia* solution. A PerkinElmer (Waltham, MA, USA) single-beam flame atomic absorption spectrometer, model 1100B, with a deuterium lamp for background correction was applied for determining the concentration of Au(III) in the solution. Working conditions for the instrument operation were selected according to recommendations of the spectrometer manufacturer.

The morphology of purified Au NPs was determined using scanning electron microscopy (SEM). A Jeol JSM-6610LVnx instrument, equipped with an Aztec Energy (UK) integrated X-ray energy dispersive spectrometer, and a CCD camera, was used for that purpose. The pressure in the SEM chamber was in the range from 10 to 270 Pa. Prior to this, Au NPs were purified as previously described. The purified Au NPs were diluted, placed on a carbon sticky tape, and evaporated. Images of samples were recorded at different magnifications. The operating voltage equal to 18–28 kV was applied. The magnification with satisfactory sharpness and resolution was from 3000 to 14,000.

The size of Au NPs, reflected by the hydrodynamic diameter, was determined by the dynamic light scattering (DLS) using a Malvern Instrument (UK) Zetasizer Nano-ZS device in optically homogeneous polystyrene cuvette. Measurements were performed using a He–Ne 633 nm laser at the detection angle of 173°. A Zetasizer software (Dispersion Technology) was applied for the data collection and the analysis. All measurements were performed at 25 °C and each data were the average of three runs with at least ten surveys. The polydispersity index (PdI) as well as the distribution of the mean size of Au NPs (histogram) was obtained directly from the Zetasizer software.

## Results and discussion

### Visual observations

**Fig. 2:**
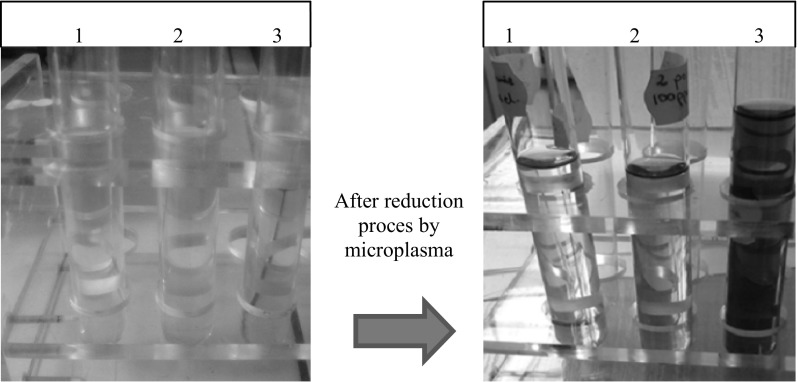
Solution of HAuCl_4_ **1** 50, **2** 100, and **3** 200 mg L^−1^ of Au before (*right*) and after (*left*) their treatment by dc-*μ*APGD system

It was found that dc-μAPGD generated between the miniature flow Ar microjet and the flowing liquid cathode is a powerful device enabled to reduce the Au precursor (present in solutions of the liquid cathode at 50, 100, and 200 mg L^−1^ of Au) in a continuous flow mode. Phenomena occurred in the discharge in the interfacial zone and the liquid likely initiated changes in the color of HAuCl_4_ solutions loaded into the microdischarge system. Accordingly, the initial yellowish color of these solutions, related to the presence of AuCl_4_^−^ ions, was turned into ruby red and bluish (see Fig. 2 in details). Mentioned changes in the color of solutions treated by dc-μAPGD pointed out that the Au NPs of different sizes were formed due in these conditions due to plasma-mediated chemical reactions and processes (Sharma et al. 2009). Au NPs were well dispersed in collected solutions and no sedimentation was observed. The temperature of solutions treated by the microdischarge did not exceed 40 °C. In addition, visual observations indicated that the intensity of the color of solutions was dependent on the concentration of the Au precursor. Accordingly, the growth of the concentration of Au ions in solutions caused an increase in their color depth. This could possibly be associated with changes in the size of Au nanostructures formed in the solution (Patel et al. 2013).

### UV/Vis measurements

**Fig. 3:**
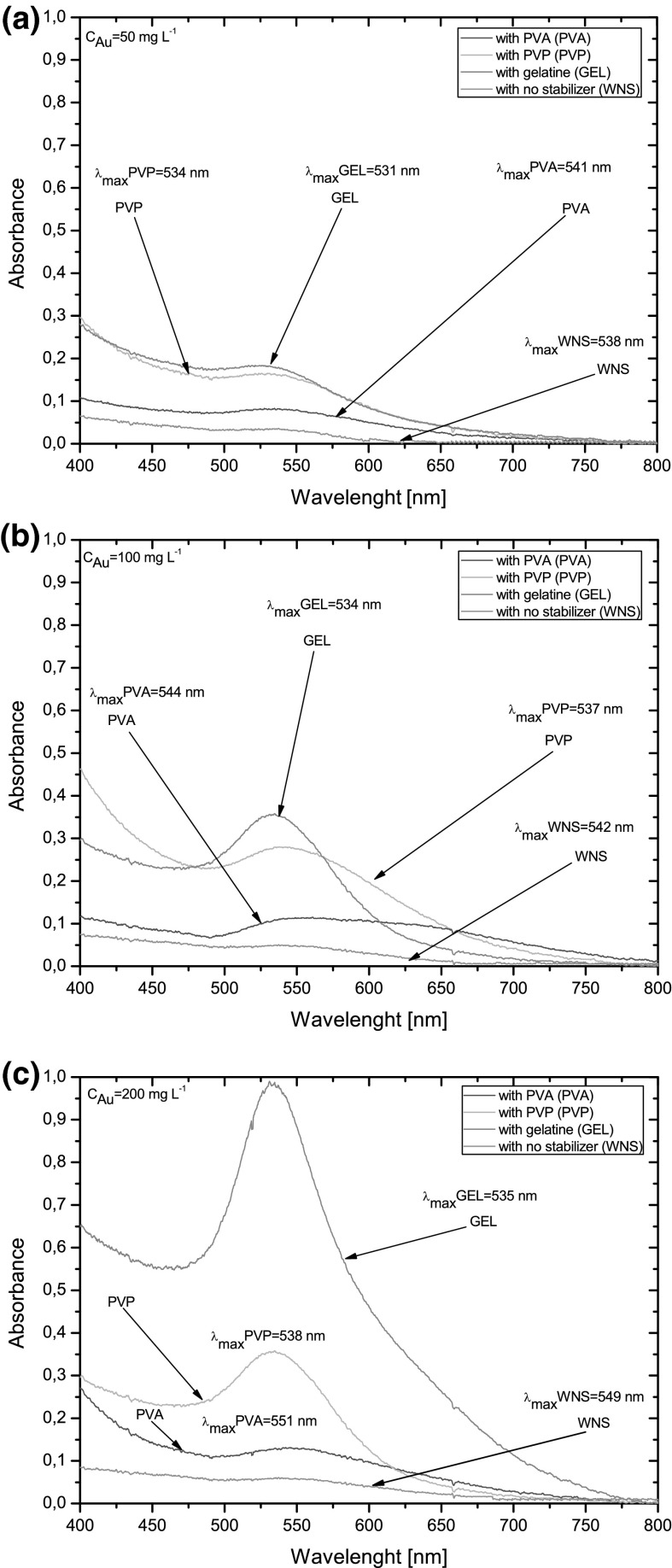
UV/Vis absorption spectra of Au NPs synthesized by dc-*μ*APGD from solution of Au containing no stabilizer (WNS) or capping agents (GEL, PVA, and PVP). The concentration of Au in loaded solutions **a** 50, **b** 100, and **c** 200 mg L^−1^

UV/Vis absorption spectroscopy was used to evaluate optical properties of Au NPs synthesized from solutions containing Au(III) ions at three different concentrations, i.e., 50, 100, and 200 mg L^−1^. In addition, the influence of three various stabilizers, i.e., GEL, PVP, and PVA, on the production of Au NPs by dc-μAPGD was compared. As can be seen from Fig. 3a–c, LSPR bands within the 520–550 nm spectral range, distinctive for nanostructures, were identified in UV/Vis absorption spectra of all solutions treated by the microdischarge. The mentioned LSPR band is acknowledged as a feature of the growth of colloidal Au NPs (Ghosh et al. 2004).

For solutions of the Au precursor with no stabilizer added (see Fig. 3a–c), very weak and broaden LSPR bands, with maximums at 538, 542, and 549 nm, respectively, for 50, 100, and 200 mg L^−1^ solutions of Au(III), were observed. The red shift of the position of the LSPR band was probably related to the growth of the mean size of Au NPs (Wang and Gunasekaran 2012). According to the Mie’s theory (Hao and Schatz 2004), larger particles exhibit a red-shifted absorbance peak. Absorbance values at the maximum of the LSPR band in these conditions were slightly increased with the increasing concentration of the Au precursor in solutions (Fig. 3a–c). In addition, it was observed that more intense LSPR bands were achieved when capping agents (GEL, PVP and PVA) were also added to solutions of the Au precursor. It was valid for all studied concentrations of Au in solutions of the liquid cathode and reflected by proportional changes in absorbance values at the maximum of the LSPR band. Accordingly, for GEL, the absorbance value of the LSPR band increased from 0.182 to 0.998 when the concentration of Au increased from 50 to 200 mg L^−1^, respectively. Concurrently, a small shift in the wavelength of the band maximum was noted, i.e., from 531 to 535 nm. Corresponding results were obtained for other stabilizers used in the same experimental conditions, i.e., PVP and PVA. In the case of PVP, the absorption at the maximum of the LSPR band increased from 0.164 to 0.356. The position of the band was also shifted, respectively, from 534 to 538 nm. Finally, using PVA as the stabilizer of Au NPs, the absorbance value of the LSPR band increased the less, i.e., from 0.082 to 0.129. The maximum position of the band in these conditions moved toward long wavelengths, i.e., from 541 to 550 nm.

Besides these measurable differences, variations in the shape of the plasmon band were noted between studied stabilizers. The shape of the plasmon band in case of solutions with added PVA was evidently different from these observed in case of GEL or PVP added into solutions of the flowing liquid cathode. In this case, low absorbance values of the LSPR band, a strong absorption of the radiation in the spectral range above 600 nm and relatively high full-widths at the half maximum (FWHMs) of the band could indicate the production of Au NPs with the worst granulometric properties. In the case of PVP and particularly GEL, the plasmon band was characterized by relatively high absorbance values at the maximum of the peak, the high symmetry, and low values of FWHMs.

Comparing all the UV/Vis absorption spectra acquired in different conditions, it was concluded that GEL was the best capping agent among all examined stabilizers for the production of Au NPs in the present work. Because the wavelength of the maximum of the plasmon band changed very little in these conditions, it was assumed that spherical Au NPs structures were formed (Link et al. 1999).

**Fig. 4:**
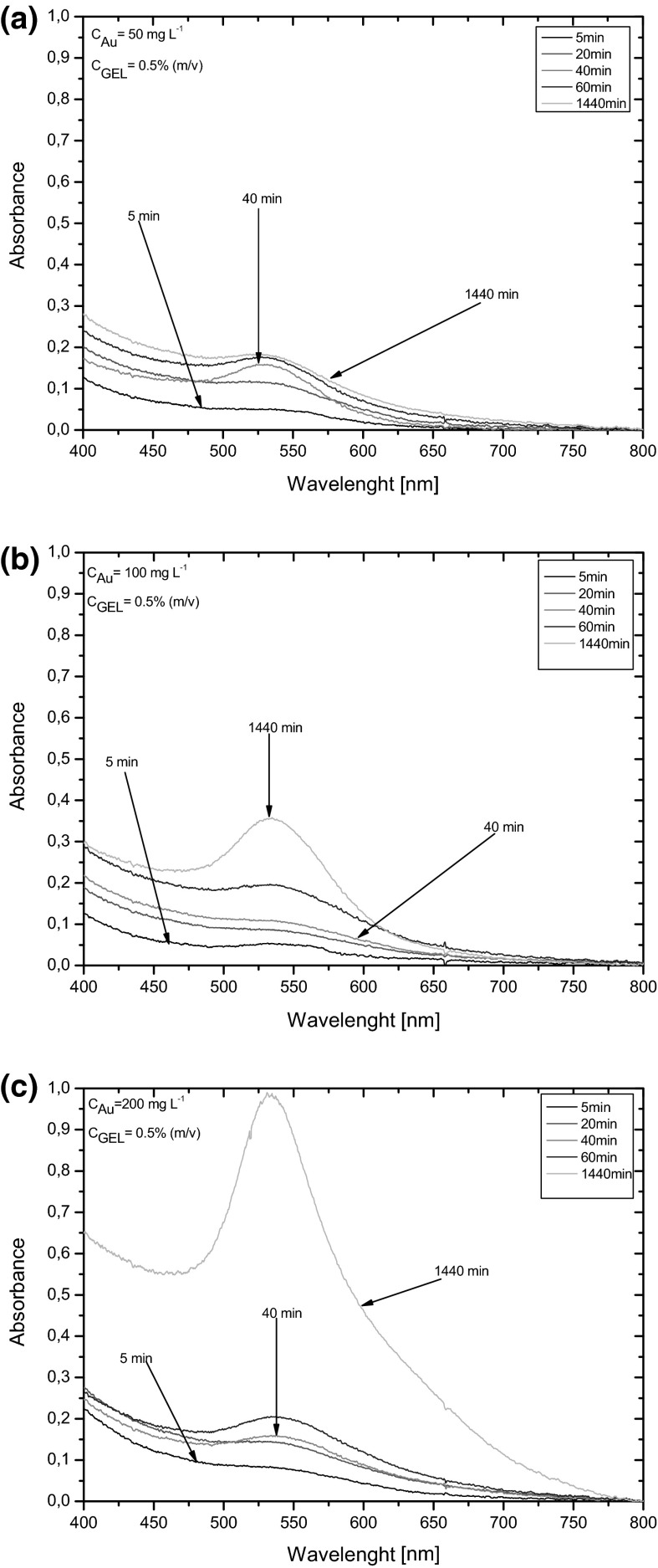
Absorption spectra of Au NPs as a function of post-treatment time for solutions containing **a** 50, **b** 100, and **c** 200 mg L^−1^ of Au and 0.5 % (m/v) GEL

Next, using GEL as the most effective anti-coagulant and anti-aggregation agent, the effect of the post-microdischarge treatment time (5, 20, 40, 60, and 1440 min), after which UV/Vis absorption spectra of solutions treated by dc-μAPGD were acquired, on the absorbance at the maximum of the LSPR band was studied. As can be seen from Fig. 4, the appearance of the LSPR band was observed directly after the microdischarge treatment. A rapid growth of Au NPs likely occurred after 40 min that was reflected by a gradual increase in absorption values of the maximum of the LSPR band. The absorbance at the maximum of the LSPR band reached the highest value at 1440 min and the same effect was observed for other concentrations of Au used in loaded solutions. Using times higher than 1440 min, absorbance values at the maximum of the plasmon band were practically unchanged. Interestingly, a similar dependence was also observed for other capping agents, i.e., PVP and PVA. The growth of the absorption of LSPR band corresponds to the progress of the reduction of Au(III) ions and the formation of Au NPs (Okitsu et al. 2005).

Since APGD generated in contact with liquid is established to be a rich source of various reactive oxygen species, e.g., H_2_O_2_, and OH, formed in interfacial and liquid phases of the discharge (Richmonds and Sankaran 2008; Jamróz et al. 2014a, b), a likely explanation of the action of GEL and the effect of the post-microdischarge treatment time was given. Accordingly, H_2_O_2_ and hydrated electrons (*e*_aq_), produced by the microdischarge, could participate in the reduction of Au(III) ions in the liquid phase to Au^0^, e.g., 2Au^3+^+3H_2_O_2_ = 2Au^0^ + 3O_2_ + 6H^+^; Au^3+^+3*e*_aq_=Au^0^. The observed increase in the absorbance of the LSPR band versus the time (up to 1440 min) suggested, however, that the microdischarge could only initiate the process of the formation of Au NPs by the production of certain substrates (e.g., H_2_O_2_, *e*_aq_) capable of reducing Au(III) ions. GEL, likely broken down into amino acids and/or simple peptides in conditions of the dc-μAPGD treatment, could continue to protect Au NPs from the agglomeration and/or the sedimentation (van Eerd et al. 2006).

The applied continuous flow mode system enabled the online production of Au NPs. Due to a relatively high exposure of solutions to the discharge (taking into account the diameter of the liquid cathode and the flow rate of solution), the system likely enhanced the production rate of Au NPs. It was also eligible to quench the nanoparticle growth, and thus the production of Au NPs with given properties was possible. Additionally, the synthesis process of Au NPs took place in controlled conditions that guaranteed a better reproducibility of the process as compared to traditional (batch) systems. Continuous flow mode systems could also enable to scale-up the production of NPs (Lin et al. 2004).

### SEM and DLS analysis of Au NPs

Due to the lowest wavelength value of the maximum of the LSPR band and the highest absorbance values observed in case of the microdischarge-assisted production of Au NPs from solutions of Au with added GEL, it was presumed that the use of this stabilizer likely enabled to synthesize Au NPs having the smallest size and the narrower size distribution. Such granulometric properties could be provided by the composition of GEL, which is a mixture of peptides and proteins containing amino acids (Wang and Gunasekaran 2012).

**Fig. 5:**
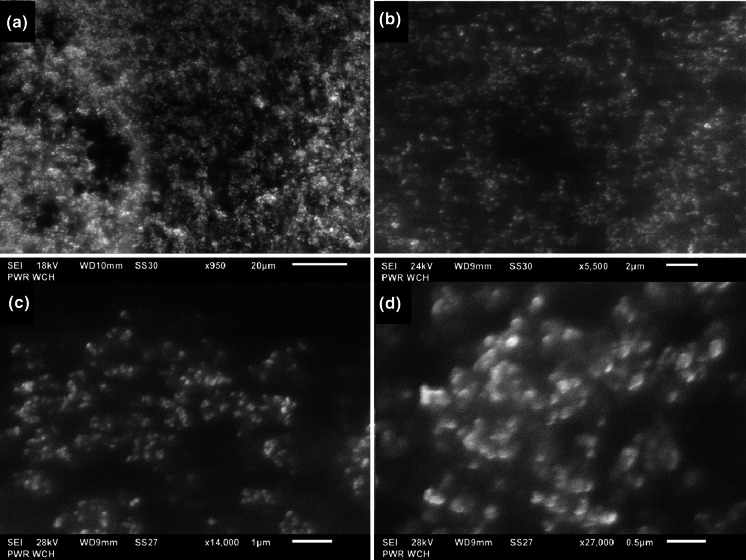
Representative SEM images of Au NPs taken with different magnification: **a** 50 ppm **b** 100 ppm, and **c**,**d** 200 ppm in the media of 0.5 % (m/v) GEL and 0.10 mol L^−1^ HCl

Scanning electron microscopy and dynamic light scattering were used to characterize the morphology of Au NPs produced in the medium of GEL. The SEM images taken for Au NPs produced from 50, 100, and 200 mg L^−1^ solutions of Au and 0.5 % (m/v) GEL are given in Fig. 5. As can be seen, it was revealed that spherical, non-aggregated, uniform in size with a very narrow size distribution mono-dispersed Au nanostructures were formed as a result of the dc-μAPGD treatment of these solutions. Due to a limited resolution of SEM, it was not possible to observe differences in the size of obtained Au NPs. The precise size determination was either difficult because grain boundaries were hardly visible. Nevertheless, SEM images revealed that the dominant feature of Au NPs is the spherical-like shape with the average diameter below 100 nm. This technique strictly confirmed the effectiveness of the microdischarge-mediated method of the synthesis of Au NPs proposed in the present work.

In addition, the production rate of Au NPs was evaluated. It was established that it was related to the precursor concentration in solutions treated by dc-μAPGD. However, the relation was not linear. At the lowest precursor concentration (50 mg L^−1^), the production rate of the synthesis of Au NPs was the lowest (2.9 mg h^−1^). A further growth of the concentration of Au in solutions between 100 and 200 mg L^−1^ resulted in an increase of the production rate of Au NPs to 3.9 and 5.7 mg h^−1^, respectively. Indeed, when the concentration of the Au precursor in solutions of the liquid cathode increased, the amount of produced Au NPs did not proportionally increase. Hence, the efficiency of the production of NPs decreased with the growth concentrations of Au in solutions. This indicated that APGD presents a certain reduction power toward the production of NPs. The rest of the unreacted Au precursor was collected along with produced Au NPs present in overflowing solutions.

**Fig. 6:**
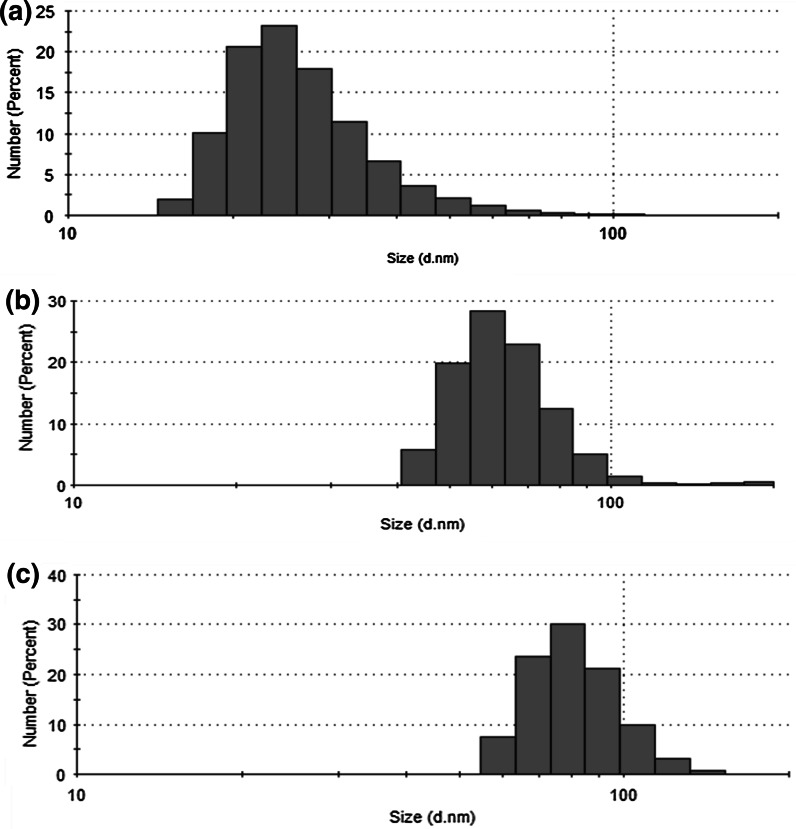
The size distribution of Au NPs obtained for a solution containing **a** 50, **b** 100, and **c** 200 mg L^−1^ of Au

To properly evaluate the size of Au NPs produced in the medium of the GEL capping agent, solutions of HAuCl_4_ treated by dc-μAPGD were also analyzed using the DLS technique. Resultant histograms of the size distribution of Au NPs are given in Fig. 6. It was established that by increasing the initial concentration of the Au precursor from 50 to 200 mg L^−1^ of Au, the mean size of Au NPs produced in these conditions was increased from 27 to 92 nm. The PdI value was found to linearly increase in these conditions from 0.296 (50 mg L^−1^ of Au) to 0.456 (200 mg L^−1^ of Au). Values of PdI also indicated a homogenous character of synthesized Au NPs.

## Conclusion

The present work proposed an original and not reported so far, the continuous flow mode production of Au NPs using discharge-mediated processes in dc-μAPGD generated in contact with the flowing liquid cathode. It was established that reactions occurring in interfacial and liquid zones of the microdischarge could possibly just initiate this process that was further spontaneously developed in the solution with time. In addition, it was found that capping agents (GEL, PVA, or PVP) could improve the production rate of Au NPs. Among studied protective agents, GEL was established to exhibit the best protective and stabilizing properties and allowed to obtain small, uniform in size, and spherical Au nanostructures. By contrast, PVP was capable of obtaining Au NPs having the most constricted size distribution, but with a larger average size than in case of GEL. Finally, PVA was the capping agent that resulted in obtaining colloidal Au NPs with the relatively worst granulometric characteristic. Nevertheless, Au NPs produced with the aid of dc-μAPGD were uniform, spherical, and of a high purity. Advantages of the proposed microdischarge-mediated continuous flow method of the synthesis of Au NPs were their specific granulometric properties and the relative simplicity of the process.
